# Clinical Partnering ‐ When Should Host Nation Patients Not be Directed to Coalition Military Hospitals?

**DOI:** 10.1111/dewb.70042

**Published:** 2026-05-20

**Authors:** Martin Bricknell

**Affiliations:** ^1^ Professor in Conflict, Health and Military Medicine, Centre for Conflict and Health Security, School of Security Studies King's College London, Strand London UK

**Keywords:** clinical partnering, Geneva Conventions, medical rules of eligibility, military ethics

## Abstract

This case commentary explores whether and when it is justifiable NOT to direct injured or ill host nation patients to international coalition military hospitals that are better equipped than host nation hospitals to meet patients' health needs in order to ensure sufficient patients for success in clinical partnering. The case discusses Medical Rules of Eligibility as a policy to determine the destination of patients who present for medical care to international military medical services. It also discusses the ethical challenges for international military healthcare workers when working as clinical partners to support capability‐building in host national medical facilities. This can be particularly difficult if it involves shared care of patients rather than didactic training. Finally, the case discusses the ethical dimensions of news reporting within medical facilities, especially if this is part of the military effort to share information about the military campaign. The case highlights ethical aspects of higher‐level military policy that impact clinical care and the importance of considering if external review of such policies is needed to ensure alignment with legal and professional ethical obligations.

## 1 Case

A military campaign is being fought in an overseas country in which host nation security forces and international military forces are working together to defeat an insurgency. The international forces have deployed their own military medical units to care for their own casualties. Local security and civilian casualties are treated in local medical facilities; however, these are of a very poor standard due to lack of personnel, equipment and money. Whilst the Geneva Conventions require casualties to be treated without discrimination with prioritisation solely on clinical need, it is international military policy (Medical Rules of Eligibility) that host nation casualties presenting to the international military medical services will normally be taken to a host nation military hospital rather than an international military hospital.

A media reporter hosted by international military forces posts a video to his news organisation's website titled ‘Coalition combat medics face tough choices’. The film follows the care of several host nation policemen injured by roadside bombs. An international helicopter medical evacuation team (MEDEVAC) takes a patient to the international military hospital against military policy. The escorting healthcare professionals state to camera that they did this because the quality of care in the international military hospital is superior to the host nation military hospital. This is in spite of the presence of a clinical partnering team of international military healthcare workers (imHCW) in the host nation military hospital. A second clip shows the helicopter waiting a long time at the host nation military hospital for an ambulance to collect a patient. You are the international senior military medical adviser responsible for the policy for directing host nation patients to the host nation military hospital. Is your policy ethical? This case example is based on a real news report posted during the NATO military campaign in Afghanistan [1].

A media report such as the one cited could adversely affect public opinion and the reputation of international military medical services if the Medical Rules of Eligibility policy was perceived to be unethical. At the same time, the international coalition health care personnel may feel uncomfortable with the ethical dimensions of working with host nation health care professionals if they are not able to follow the same treatment protocols used in the coalition military hospital. Finally, the host nation military partners may feel offended if they perceive that their health care system is being criticised by their international partners.

## 2 Commentary

This case builds upon a previously published Case Commentary that discussed ‘How Should Access to Military Health Care Facilities be Controlled in Conflict?’ [2]. That Case Commentary focussed on the responsibility of a commander of a military field hospital to determine how his hospital should work with other local medical units in meeting the needs of host nation civilians during a humanitarian emergency. It identified the conflicting legal and ethical principles that need to be balanced in the determination of Medical Rules of Eligibility for different populations who might receive health care from international military health care facilities or might be directed to local facilities. The previous Case Commentary discussed the practical limitations of the principle of non‐discrimination except on grounds of clinical urgency. It emphasised the responsibility of sovereign states to meet the health care needs of their citizens and armed forces. In this Case Commentary, coalition military forces are conducting military operations alongside host nation security forces personnel in order to transition responsibility to locally‐led security forces. The partnering programme includes a ‘train‐and‐advise’ mission in which imHCWs are embedded as clinical partners within host nation military health care facilities. In accordance with your policy, host nation health care personnel should develop their skills by caring for their host nation patients with support from their imHCW clinical partners. However, it is highly likely that the clinical outcomes will be worse than for those patients receiving care in the coalition health care system.

The policy on Medical Rules of Eligibility is likely to be highly sensitive. This case examines whether it is ethical to discriminate against host nation casualties compared to coalition forces on grounds other than clinical need by sending them for medical care in a health system that is known to be inferior to that provided to international forces. It illustrates a tension between ‘individual‐level’ and ‘system‐level’ beneficence for patient care, mirroring the tension between clinician level decision‐making and egalitarian justice in the allocation of resources at a public health level. In this case, there was already a substantial difference between international and local funding for the host nation public health system compared to the military health system and clear disparity in access to care between urban and rural settings. There is a clearly identifiable risk of different clinical outcomes between different elements of the whole health system for the patient (in both morbidity and possibly mortality) between coalition medical care and host nation care. This has been implicitly accepted in the policy on Medical Rules of Eligibility. In reality, most health systems have measurable differences in likely clinical outcomes between publicly funded health care and non‐publicly funded healthcare paid for by insurance or personal wealth. However, there is always an obligation to provide emergency care pending transfer to the healthcare system that the patient is entitled to access. In this case, the host nation patients are receiving emergency care from coalition forces and are being rapidly transported direct to the host nation military hospital. It could be argued that the clinical risk for individuals from not being directly admitted to the international coalition hospital is being mitigated through a programme of clinical partnering in the host national hospital by imHCWs.

## 3 Ethical Issues From Clinical Partnering

Military health care practitioners may experience unfamiliar ethical challenges when undertaking Global Health Engagement (GHE) missions. The breadth of these challenges in Defence Engagement (Health) (DE(H), the British term for GHE) have been categorised at the Strategic, Operational and Tactical levels [3]. ‘Embedded partnering’ with host nation security forces would be part of the strategic plan to transition security responsibility and capacity from international partners to the sovereign government. Whilst the decision whether or not to take a specific host nation patient to a coalition medical facility might seem to be a minor tactical issue, the policy is an operational level issue, and adverse publicity about the policy could have strategic implications. This policy of directing host nation patients to the host nation military hospital could be ethically justified if the intention is to achieve ‘beneficence’ at a system level even if there is discrimination on grounds other than clinical need in the care for individual patients.

It is likely that international military medical staff will have assessed the status of the health care services that support the host nation security forces. This may have led to a programme to invest in health care infrastructure and equipment. Such health sector capacity development programmes are highly dependent on a parallel training and education programme to increase the number and competence of the host nation health care workers [4]. This may be achieved by embedding imHCWs as clinical partners into the host nation military health care facilities to help share knowledge, skills and competence in clinical procedures. However, it is necessary for these facilities to receive patients in order to improve the skills of their clinicians. This will inevitably require adjustments of the clinical expectations of the imHCWs to the practical realities of the competence, capacity, resources, motivations, culture and ethical foundations of host nation health care practitioners [5]. These differences may cause ethical concerns about practicing in an unfamiliar environment without the resources they would expect. At a system level, these ethical tensions could cause moral injury to imHCWs if they are not fully prepared for their role.

The arrangements for embedded imHCWs will need to be agreed at a senior level between international and host nation medical leaders. It will be necessary to confirm that clinical responsibility is held by the host nation clinical leadership with the imHCWs in a supporting role. Where interpreters are required, they should support this principle rather than enabling an imHCW to provide direct patient care [6]. There will need to be an agreed process for imHCWs to report clinical concerns to both the host nation and international medical leadership.

These imHCWs will need to be competent clinical educators in their own right. It is important that they receive orientation to their specific role, including discussions on the realities of clinical care in a low resource environment, and ethical dilemmas. Professional collaboration between imHCWs and host nation HCWs should also include confidence building measures to allow progressive transfer of host nation patients of increasing clinical severity from international health care facilities to the local military hospitals. This may include direct admission from international helicopters and ambulances. Each step should be rehearsed covering both clinical and organisational procedures. These confidence building measures might also involve inward visits by personnel from the international military hospital and medical evacuation crews to the host nation hospital so that their views on the competence of the host nation military hospital are based on facts rather than unsubstantiated rumour. The policy for direct evacuation to the host nation military hospital should include delegated authority to divert to the international military hospital in extremis to ensure compliance with ethical obligations to provide emergency care [7].

## 4 Specific Considerations

It is not clear whether the rationale for the policy on Medical Rules of Eligibility was fully communicated to clinicians or had been subject to an independent ethical review. It is necessary to ensure that the policy documents contain the full information for clinicians to understand the background and considerations in the formulation of the documents. It might also be necessary to develop a bespoke training package on this topic for all imHCWs. Senior military medical leaders need to consider whether to refer policy decisions with significant ethical ramifications for external review in order to protect them from accusations of professional dereliction of duty.

This case also exposes an ethical conundrum over patient privacy and the use of clinical images from health care settings for media purposes without the consent of patients. The International Committee of the Red Cross (ICRC) recommends that during conflict the wounded and sick should be shielded from media scrutiny [8]. The World Medical Association Declaration of Lisbon on the rights of the patient states: the patient's dignity and right to privacy shall be respected at all times in medical care and teaching, as shall his/her culture and values' [9]. It would seem that it was ethically unacceptable for a health care professional to allow a news reporter to capture images of a patient during their care without explicit consent. Health care professionals should also be very wary of making statements about the quality of care in other health care institutions unless they have clear and citable evidence. They should also consider the implications of making such statements in the public domain.

In this specific case, there should be a review of the clinical cases presented in the news report to confirm the appropriateness of MEDEVAC team's decision to override policy and the criticisms of the reception of patients into the host nation military hospital. This process can be designed as a confidence‐building measure to improve understanding of respective clinical capabilities. It might also further empower the imHCW clinical partners in their relationships with host nation health care staff.

The appropriateness of allowing a news team to record patients in a clinical environment should be discretely reviewed. Many observers would consider this to be unethical, so the arrangements for the authorisation of the news team should be checked. International military health care professionals should be reminded of their duty to protect the dignity and privacy of their patients. Finally, a senior medical leader might consider meeting the news team to put their report into context and explain that it will not be possible for them to take clinical images of patients in the future because of ethical concerns over patient privacy.

## 5 Conclusion

The discussion explains how the policy of direct transfer of host nation patients to host nation health care facilities can be ethical even if it is known that the clinical outcomes are likely to be worse than direct admission to an international military hospital. However, the purpose of the policy should be explicitly recorded and confirmed with senior military and medical leaders. This might include an external ethical review in order to protect the signatories from investigation by professional regulatory authorities.

Clinical partnering is one of the medical lessons from operations in Afghanistan and led to the concept of DH(E) [10, 11]. The commentary has highlighted some of the ethical challenges of clinical partnering and the importance of trust‐building measures between clinical teams. It is necessary to ensure that members of clinical partnering teams are properly trained for their role and have had specific education in the likely ethical challenges that they may face.

This case commentary has closed by re‐emphasising the duty of health care professionals to protect the dignity and privacy of their patients by preventing recording of clinical care without the patient's explicit consent. This is especially relevant when the provision of health care might be considered part of the communication policy for the military campaign to offset some of the negative aspects of conducting military operations.

## Author Contributions

M.B. both conceived and drafted this paper.

## Funding

The author has nothing to report.

## Ethics Statement

The author has nothing to report.

## Consent

The author has nothing to report.

## Conflicts of Interest

M.B. was Surgeon General of the UK Defence Medical Services 2018‐2019. The author declares no conflicts of interest.

## Data Availability

Data sharing not applicable to this article as no datasets were generated or analysed during the current study.

## References

[1] “US Combat Medics Face Tough Choices in Afghanistan” Al Jeezera December, 21 2009 at: https://www.youtube.com/watch?v=kKHVCASh6ME.

[2] M. Bricknell , D. Whetham , R. Sullivan , and P. Mahoney , “How Should Access to Military Health Care Facilities Be Controlled in Conflict?,” AMA Journal of Ethics 24, no. 6 (2022): 472–477. 10.1001/amajethics.2022.472 35713914

[3] M. Bricknell and J. Kelly , “Ethical Tensions in Delivering Defence Engagement (Health),” BMJ Military Health 170, no. e1 (2024): e36–e39. 36787909 10.1136/military-2022-002318

[4] M. C. Bricknell and D. Thompson , “Roles for International Military Medical Services in Stability Operations (Security Sector Reform),” Journal of the Royal Army Medical Corps 153, no. 2 (2007): 95–98. 17896536 10.1136/jramc-153-02-04

[5] E. K. Burkett , B. Neese , and C. Y. Lawrence , “Developing the Prototype Embedded Health Engagement Team,” Military Medicine 185, no. Suppl 1 (2020): 549–553. 32074376 10.1093/milmed/usz207

[6] M. C. Bricknell , “Reflections on Medical Aspects of ISAF IX in Afghanistan,” Journal of the Royal Army Medical Corps 153, no. 1 (2007): 44–51. 10.1136/jramc-153-01-11 17575877

[7] M. C. Bricknell and E.‐J. Grigson , “International Military Medical Engagement With the Indigenous Health Sector – Afghan Security Forces Medical Services,” supplement, Journal of the Royal Army Medical Corps 157, no. 4, S2 (2011): S468–S472.

[8] R. Coupland and A. Brietegger , “The Responsibilities of Health‐Care Personnel Working in Armed Conflicts and Other Emergencies” International Committee of the Red Cross, accessed March 20, 2025, https://www.icrc.org/en/publication/4104-health-care-danger-responsibilities-health-care-personnel-working-armed-conflicts.

[9] “Declaration of Lisbon on the Rights of the Patient”. World Medical Association, April 2015, accessed March 20, 2025, https://www.wma.net/policies-post/wma-declaration-of-lisbon-on-the-rights-of-the-patient/.

[10] M. C. M. Bricknell and M. Nadin , “Lessons From the Organisation of the UK Medical Services Deployed in Support of Operation Telic (Iraq) and Operation Herrick (Afghanistan),” Journal of the Royal Army Medical Corps 163, no. 4 (2017): 273–279. 28062527 10.1136/jramc-2016-000720 PMC5629939

[11] S. Tallowin , D. N. Naumann , and D. M. Bowley , “Defence Health Care Engagement: A Uk Military Perspective to Improve Health Care Leadership and Quality of Care Overseas,” Journal of Health Care Leadership 13 (2021): 27–34. 10.2147/JHL.S224906 PMC7854361 33542672

